# Preparation Methods of Polypropylene/Nano-Silica/Styrene-Ethylene-Butylene-Styrene Composite and Its Effect on Electrical Properties

**DOI:** 10.3390/polym11050797

**Published:** 2019-05-04

**Authors:** Mingze Gao, Jiaming Yang, Hong Zhao, Hui He, Ming Hu, Shuhong Xie

**Affiliations:** 1 Key Laboratory of Engineering Dielectric and its Application, Ministry of Education, Harbin University of Science and Technology, Harbin 150080, China; gaomingze00@163.com (M.G.); angleman666@163.com (H.H.); 2 Zhongtian Technology Submarine Cable Co., Ltd., Nantong 226000, China; hum@chinaztt.com (M.H.); xiesh@chinaztt.com (S.X.)

**Keywords:** polypropylene, nano-SiO_2_, SEBS, space charge, DC breakdown

## Abstract

Compared with traditional insulation materials, such as cross-linked polyethylene (XLPE), polypropylene (PP) is famous for its better recyclable and thermal properties, as well as its good electrical performance. However, the problem of poor impact strength has restricted the application of pure PP in high-voltage, direct current (HVDC) cables. In this paper, styrene-ethylene-butylene-styrene block copolymer (SEBS) was used as a toughening filler, and nano-SiO_2_ was expected to improve the electric properties of the nano-composite. By controlling the masterbatch system, the dispersion characteristics of nano-SiO_2_ in the ternary composite system were changed. When PP/SiO_2_ was used as the masterbatch and then blended with SEBS, nano-SiO_2_ tended to disperse in the PP phase, and the number of nano-particles in the SEBS phase was lower. When PP/SEBS was used as the masterbatch, nano-SiO_2_ was distributed in both the PP phase and the SEBS phase. When SEBS/SiO_2_ was used as the masterbatch, nano-SiO_2_ tended to be dispersed in the SEBS phase. The different dispersion characteristics of nano-SiO_2_ changed the crystallization and mechanical properties of the ternary composite system and produced different electrical performance improvement effects. The results of our experiment revealed that the space charge suppression capability was positively correlated with the direct current (DC) breakdown strength improvement effect. Compared with the DC performance of 500 kV commercial XLPE materials, the self-made PP-based ternary composite system has better space charge suppression effects and higher DC breakdown strength. When nano-SiO_2_ was more dispersed in the PP phase, the space charge improvement effect was best. When the nano-SiO_2_ particles were more dispersed in the SEBS phase, the expected electrical property improvement was not obtained. Scanning electron microscopy showed that the nano-SiO_2_ particles in the SEBS phase were more dispersed at the interface than in the SEBS matrix, indicating that the nano-particles were poorly dispersed, which may be a reason why the electrical properties of the composite system were not significantly improved.

## 1. Introduction

In recent years, with the introduction of concepts such as the super grid and the global energy interconnection, the demand for high-voltage power cables has increased. Intercontinental power interconnection over long distances across the sea needs to be achieved with high-voltage DC (direct current) cables [1]. The application of cross-linked polyethylene (XLPE) to high-voltage DC cables, however, needs to solve the problem of space charge accumulation [2]. Based on chemically pure technology, some manufacturers have effectively suppressed the space charge by reducing the content of chemical impurities in XLPE and have successfully developed ±500 kV high-voltage, direct current (HVDC) cable XLPE insulation material [3]. Japanese researchers filled XLPE with nano-particles, which also obtained significant space charge suppression effects, and successfully developed a ±500 kV high-voltage DC cable [4]. Although XLPE insulation has been successfully applied to HVDC cables, it is a thermoset material that cannot be recycled. This is problematic, because the decommissioned materials are extremely difficult to recycle and put tremendous pressure on the environment. In order to find high-performance, recyclable, environmentally friendly polymer insulation materials, thermoplastic polymer materials have received increasing attention in recent years [5].

Polypropylene (PP) material has a high melting temperature and excellent electrical and mechanical properties [6] and has been widely used in power capacitors and other fields. It can also be used as an environmentally friendly cable insulation material to replace XLPE materials [7]. However, its poor low-temperature impact strength makes it impossible to directly apply to cable manufacturing [8,9]. The poor low-temperature impact strength of PP can be significantly improved by filling with thermoplastic elastomer, but the addition of the elastomer increases the space charge and significantly reduces the breakdown strength of PP [10,11]. In order to solve this problem, the electrical properties of PP/elastomer composites can be improved by filling with nano-additives. Zha reported that adding nano-ZnO to PP/styrene-ethylene-butylene-styrene block copolymer (SEBS) can significantly reduce the space charge and increase the DC breakdown strength of the materials [12]. Chi added nano-SiO_2_ to PP/polyolefin elastomer, which also reduces the space charge of the composite [13]. However, the above studies paid less attention to the dispersion state of the nano-particles in the composite. From scanning electron microscope images provided by Zha and Chi, the elastomer phase and the PP phase cannot be clearly distinguished, and the dispersion information of the nano-particles in different phases cannot be obtained. In a ternary composite system composed of nano-particles, the elastomer phase, and the PP phase, when the nano-particles are more dispersed in the PP phase or more dispersed in the SEBS phase, different electrical property improvement effects are produced. 

In this paper, three different kinds of masterbatch were prepared by selecting a combination of two materials from among nano-SiO_2_ particles, PP, and SEBS and then blending them with the remaining materials to prepare ternary composites. By changing the masterbatch system, the nano-particles form three different distribution states, which are more dispersed in the PP phase, more dispersed in the SEBS phase, and evenly distributed in the two phases. By studying the prepared materials, the influence of the distribution of the nano-particles on the crystallization, mechanics, and electrical properties of the ternary composites was further analyzed. Compared with the DC dielectric properties of 500 kV commercial XLPE insulation materials, the DC insulation performance of the self-made PP-based ternary composite system was evaluated.

## 2. Materials and Methods 

### 2.1. Materials

The matrix of isotactic PP (iPP, Sinopec, Beijing, China) purchased by Sinopec. SEBS (G1652EU) was supplied by Kraton Corporation (Belpre, OH, America). Nano-SiO_2_ (AEROSIL R812S) with an average diameter of 20 nm was obtained from Evonik Industries AG (Frankfurt, Germany). Nano-SiO_2_ was surface treated with hexamethyldisilazane; compared with other surface modifiers, such as 3-aminopropyltriethoxysilane, it can coat the surface of nano-particles with a dense alkane molecular structure, which significantly reduces the polarity and improves the dispersion of nano-SiO_2_. The comparative material, XLPE, was a commercial ±500 kV high-voltage DC cable material. The antioxidant (Irganox 1010, Dongguan shanyi plastic co. LTD, Dongguan, China) was blended into all the samples at the beginning of the melting process to avoid degradation. PP, SEBS, and nano-SiO_2_ were dried in a vacuum oven at 80 °C for 24 h before preparation. The weight ratio of PP/SEBS/nano-SiO_2_ was 75:25:1. All the samples melted at 190 °C and 60 rpm by using an internal mixer, and PP, SEBS, and nano-SiO_2_ were melted respectively for 3, 3, and 15 min. The preparation process was as follows. First, PP and SEBS were separately melt-blended into the internal mixer to prepare masterbatch 1, which we called PP/SEBS, masterbatch 2 was prepared by blending PP with nano-SiO_2_, and masterbatch 3 was blended by using SEBS and nano-SiO_2_. Second, the nano-composites of the PP, SEBS, and nano-SiO_2_ ternary systems were prepared by using three masterbatches, respectively. Nano-SiO_2_ was blended into masterbatch 1, SEBS was added into masterbatch 2, and PP was melted into masterbatch 3, according to different blending orders. We referred to these three nano-composites as PP/SEBS/SiO_2_, PP/SiO_2_/SEBS, SEBS/SiO_2_/PP, respectively, and the blending order and abbreviations of the composites are shown in Table 1. PP samples were pressed to the required thickness with compression molding at 190 °C, and XLPE samples were placed in a mold and heated, first to 110 °C at 2 × 10^6^ Pa pressure to melt and then to 175 °C at 15 × 10^6^ Pa to crosslink the polymer.

### 2.2. Sample Preparation for Scanning Electron Microscope (SEM) Observations

All the samples were pressed to 1 mm thickness and fractured by liquid nitrogen at low temperature. The fractured surfaces of the samples were then immersed for 7 h in the solution recommended by M. Aboulfaraj for the observation of spherulitic structures in PP [14]. The formula is 1.3 wt % potassium permanganate, 32.9 wt % concentrated H_3_PO_4_, and 65.8 wt % concentrated H_2_SO_4_. This permanganic acid preferentially etches the amorphous part of the polymer in the spherulites, in such a way that the lamellae then appear clearly. This method proved particularly useful in the case of PP. Subsequently, the specimens were carefully washed with detergent, which was a mixture of concentrated sulfuric acid water and hydrogen peroxide with a volume ratio of 2:7:1 [15]. Then, all the fractured surfaces of the samples were sputtered with a very thin layer of gold in order to eliminate any undesirable charge effects during the SEM observations. Field emission scanning electron microscopy (Hitachi SU8020, Tokyo, Japan) was used to observe the microscopic morphology of the samples.

### 2.3. Thermal Properties

Crystallization and melting curves were measured by differential scanning calorimetry (DSC, DSC822e, Mettler-Toledo International, Inc., Switzerland). A specimen weighing approximately 5 mg was heated to 200 °C at a rate of 20 °C/min and kept at that temperature for 2 min to eliminate thermal history. Then, it was cooled to 30 °C at the rate of 10 °C/min and kept at that temperature for 2 min to obtain its crystallization curve. Finally, the specimens were heated to 200 °C again at a rate of 10 °C/min to record their melting curves. The crystallinity (*X_c_*/%) was calculated from the DSC result by Equation (1).

(1) $$X_{c}=\frac{\Delta H_{m}}{(1-x)H_{0}}\times 100\%$$

where Δ*H_m_* (J/g) was the melting enthalpy; *H*_0_ was the theoretical melting enthalpy of the completely crystallized form, and *H*_0_ was 209 J/g for the isotactic PP [16]. *x* was the mass fraction of the inorganic.

### 2.4. The Dynamic Mechanical Properties

The dynamic temperature relaxation spectrum was tested by the dynamic mechanical analysis (DMA) method using DMAQ800 manufactured by TA instruments (New Castle, DE, USA). The selection mode was the tensile mode, in which the target amplitude was 15 μm, the frequency was 1 Hz, the static force was 0.375 N, and the dynamic force was 0.3 N. The sample was cuboid with a length, width, and thickness of 15 mm, 6 mm, and 1 mm, respectively. The sample was first cooled to −80 °C for 5 min, and then, the elastic modulus *E’*, the loss modulus *E”*, and the loss factor tan δ from −80 °C to 160 °C were measured at a linear heating rate of 3 °C/min.

### 2.5. Thermally Stimulated Depolarization Current

The charge trap characteristic was tested using the thermally stimulated depolarization current (TSDC) method. The prepared samples were electrically polarized by applying 40 kV/mm DC high voltage for 1 h in a vacuum environment at 60 °C. The liquid nitrogen was then used for rapid cooling the samples to −80 °C so that all kinds of charge carriers had been “frozen”, after which the DC high voltage was removed and the samples were short-circuited for about 10 min. After that, the sample was heated linearly under a constant heating rate of 3 °C/min, and the short-circuit current was measured using a 6517B electrometer (Keithley Instrument Inc., Cleveland, OH, USA).

### 2.6. DC Breakdown Test 

A DC breakdown test was undertaken on the film samples. The PP samples were tested at room temperature, 90 and 120 °C, and the XLPE samples were tested at room temperature, 50, 70, and 90 °C. Samples with thickness of 100 *μ*m were placed between two electrodes. The samples were immersed in transformer oil to prevent surface flashover, and the voltage ramp was 2 kV/s.

### 2.7. Space Charge Measurement 

The space charge distribution within the samples under DC electric field was measured using the pulsed electro-acoustic (PEA) system at room temperature. The space charge measurement was performed with a pulsed electro-acoustic (PEA) system produced by Shang Hai Xiangtie electromechanical device Co., Ltd., Shanghai, China. All the samples were kept under a 40 kV/mm DC electrical field for 1 h and then short-circuited for 1 h. Space charge profiles were recorded at various times for analysis. 

## 3. Results

### 3.1. Morphology

**Figure 1:**
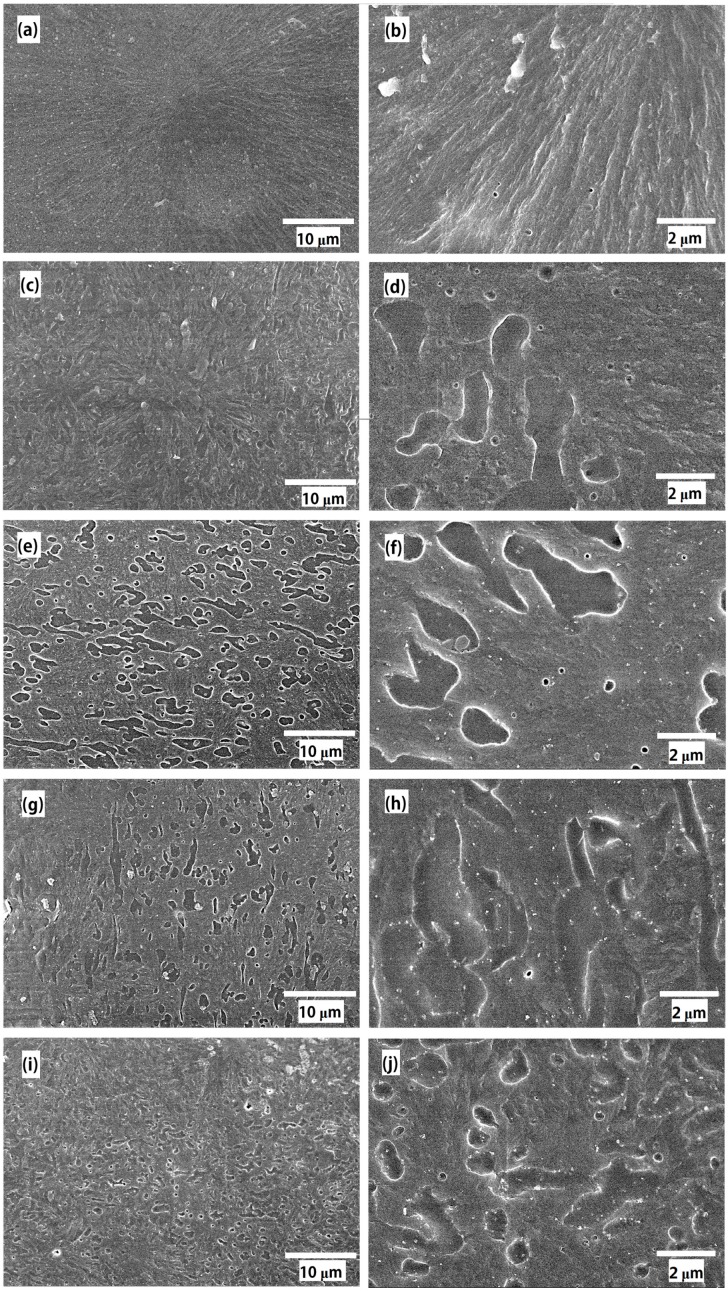
SEM picture of (**a**) PP, (**b**) PP, (**c**) PP/SEBS, (d) PP/SEBS, (**e**) PP/SiO_2_/SEBS, (**f**) PP/SiO_2_/SEBS, (**g**) PP/SEBS/SiO_2_, (**h**) PP/SEBS/SiO_2_, (**i**) SEBS/SiO_2_/PP and (**j**) SEBS/SiO_2_/PP with different magnification.

The SEM images of all the samples are shown in Figure 1. It can be seen from the images that the structure of the PP crystal was regular, and the spherulite diameter was about 100–120 μm. Many scholars have also confirmed the morphological structure of PP crystals [17]. The high-field electrical properties of PP, particularly the breakdown strength, are associated with the features of spherulites [18]. 

Because SEBS material contains polystyrene block copolymer and the material is amorphous in the aggregation state, PP and SEBS cannot be homogeneous blends [19]. In Figure 1c, the “voids” in the PP/SEBS composites are the etched SEBS phase. The “sea-island” structure distribution with SEBS as the “island” phase and PP as the “sea” phase were presented in the PP/SEBS composite [20]. The white bright spots in Figure 1f,h,j are the nano-SiO_2_ particles. Due to the different preparation methods, in Figure 1f,h,j, it was found that nano-SiO_2_ in the PP/SiO_2_/SEBS sample was mostly dispersed in the PP phase and a few in the interface between PP and SEBS. In PP/SEBS/SiO_2_ nano-composites, both the PP phase and the SEBS phase had nano-SiO_2_ particles, while in the SEBS/SiO2/PP sample, nano-SiO_2_ was concentrated in the PP and SEBS interface, partly in the SEBS phase and a few in the PP phase. The dispersion of nano-SiO_2_ in the PP phase was relatively uniform, but the distribution of nano-SiO_2_ in the SEBS phase was not uniform.

In the large field of view in Figure 1c,e,g,i, it was observed that there was a spherulite morphology in the PP/SEBS composite, and the crystal structure was smaller and looser than in PP. It was also found that SEBS was distributed along the direction of the lamella growth. The dispersion state of SEBS in PP/SiO_2_/SEBS and PP/SEBS/SiO_2_ was similar, while SEBS in SEBS/SiO_2_/PP was significantly smaller. This indicates that nano-SiO_2_ in the SEBS phase caused SEBS to form small islands and facilitated the SEBS distribution in the PP matrix. One possible mechanism was that the surface of the nano-SiO_2_ particles modified by hexamethyldisilazane was coated by dense alkane molecules, which reduced the polarity of nano-SiO_2_. It can be seen from the SEM that the dispersion of nano-SiO_2_ in the non-polar PP phase was better than that in the weak polar SEBS phase, and a large number of nano-SiO_2_ particles were distributed in the interface between SEBS and PP, which improved the compatibility between SEBS and PP, showing that the dispersion of SEBS in PP had been improved.

### 3.2. Thermal Properties

**Figure 2:**
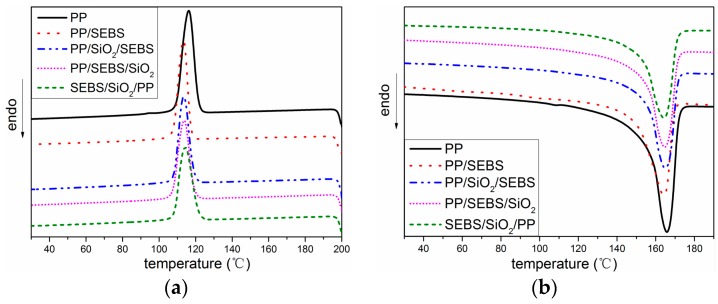
Heating and cooling curves of differential scanning calorimetry (DSC) for PP and its composite. (**a**) crystallization process; (**b**) melting process.

The DSC curves of all the samples are shown in Figure 2, and the crystallization temperature (*T*_c_), melting temperature (*T*_m_), and crystallinity (*X*_c_) are summarized in Table 2.

When SEBS was added into PP, the *T*_c_ and *T*_m_ of PP/SEBS were reduced compared with that of PP. A similar result was reported by Jyotishkumar; a low concentration of SEBS resulted in little change in the *T*_c_ and a higher *T*_m_ of the polymer, but when the amount of SEBS increased to 20%, the *T*_c_ and *T*_m_ of composite were decreased [21]. When SEBS was less filled, SEBS acted more as a nucleating agent to promote the crystallization, but when the concentration of SEBS increased to a larger extent, more SEBS entangled with the molecular chain of PP, suppressing the crystallization process of PP and causing the decrease of the *T*_c_. As shown in Figure 1, SEBS made PP spherulites smaller, and the structure became looser. This phenomenon allowed the PP crystal to melt at a lower temperature, so the *T*_m_ peak of PP/SEBS was lowered. 

As shown in Table 2, the differences in the *T*_c_ and *T*_m_ between the nano-composites and PP/SEBS were very small. Many studies indicated that nano-particles cause heterogeneous nucleation and promote the formation of crystals [22,23]. However, there was no obvious change in the *T*_c_ and *T*_m_ of nano-composites in this paper. One possible reason was that the content of nano-SiO_2_ was only 1 phr (parts per hundreds of resin); thus, nano-SiO_2_ did not produce a significant change in the *T*_c_ and *T*_m_ of the nano-composite. 

### 3.3. The Dynamic Mechanical Properties 

**Figure 3:**
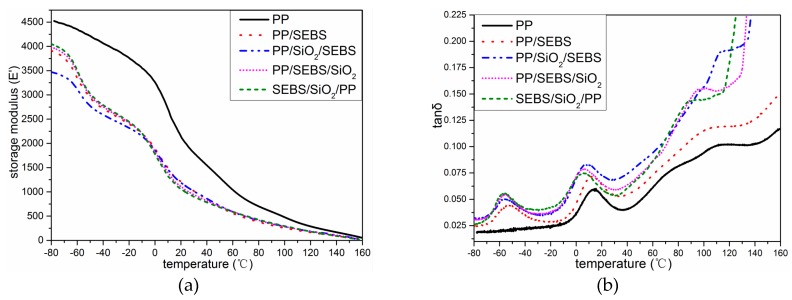
The dynamic mechanical analysis (DMA) pattern of PP and its composites. (**a**) Storage modulus (*E*’); (**b**) loss factor (tan δ).

The dynamic mechanical spectra of PP, PP/SEBS, and the three nano-composites are illustrated in Figure 3. From the change in the storage modulus (*E*’) of the different materials in Figure 3a, it can be seen that the *E*’ of the composites decreased after SEBS was added in the whole temperature spectrum, and *E*’ of the PP/SiO_2_/SEBS nano-composite had a significantly lower E’ in the temperature range of −80 to −20 °C. With the increasing of the temperature, the *E*’ of all the samples gradually decreased. It was reported that the glass transition temperature of PP was about 10 °C [24]; in the temperature spectrum of the loss factor (tan δ) in Figure 3b, the loss peak appeared at this temperature. Therefore, in the temperature range of −80 to −20 °C, the molecular chains of PP in the crystal region and the amorphous region were in a frozen state, and the E’ of the composites were mainly affected by the SEBS phase. In the PP/SiO_2_/SEBS nano-composite, nano-SiO_2_ was more dispersed in the PP phase, which induced the formation of smaller-scale spherulites, so much SEBS dispersed in the interface of PP spherulite and the E’ of PP/SiO_2_/SEBS nano-composites were more significantly reduced. As the temperature gradually increased and the lamella gradually melted, the increasing SEBS surrounded by spherulites began to decrease *E*’, and then, the *E*’ of all the samples were not much different at high temperatures.

From the change in the loss factor shown in Figure 3b, it was determined that the loss factor of the composite system increased after filling with SEBS compared with PP. After the addition of nano-SiO_2_, the loss factor of the nano-composite further increased. This was due to the entanglement of SEBS and nano-SiO_2_ with PP molecules, and the increase in friction during mechanical relaxation led to an increase in internal friction. At the same time, it was found that the mechanical relaxation peak of the PP/SiO_2_/SEBS nano-composite near 120 °C, which corresponded to the structural relaxation generated when the PP crystal region melts, was higher than those of other material systems, because the nano-SiO_2_ was more dispersed in the PP phase. Therefore, the biggest loss was produced in PP/SiO_2_/SEBS. The loss factor temperature spectra of the three nano-composites were complicated and involved various mechanisms, such as the crystal morphology change and interface zone action; thus, the specific influence needs further study.

### 3.4. Space Charge Distribution

**Figure 4:**
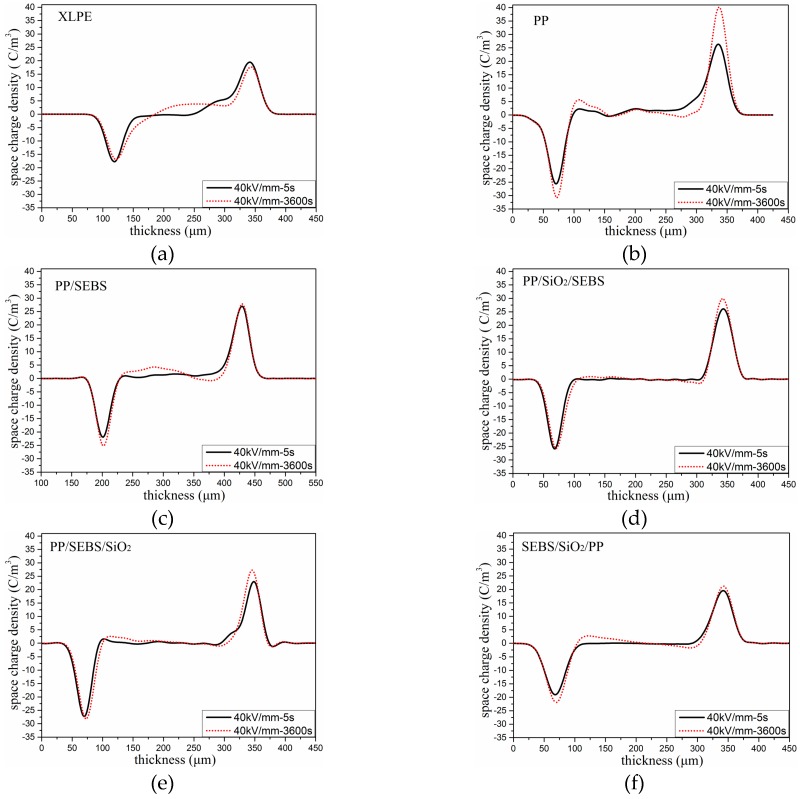
Space charge distribution under 40 kV/mm. (**a**) XLPE; (**b**) PP; (**c**) PP/SEBS; (**d**) PP/SiO_2_/SEBS; (**e**) PP/SEBS/SiO_2_; and (**f**) SEBS/SiO_2_/PP.

The space charge distributions of all the samples under a polarization electric field are illustrated in Figure 4. It can be seen from Figure 4a that a significant space charge distribution appeared in the XLPE after the electric field was applied. As the polarization time increased, the space charge migrated inside the sample. Different from XLPE, as shown in Figure 4b, when the polarization time was 5 s, there was no obvious space charge distribution in PP. When the polarization time reached 3600 s, a large amount of heterocharge accumulation appeared in the PP samples. With the addition of SEBS into PP, a large amount of heterogeneous space charge was found near the two electrodes. The amount of charge increased the longer the increasing voltage was applied, and more charges moved to the interior in PP/SEBS. With the addition of nano-SiO_2_, the space charge was decreased, and a small amount of space charge was accumulated in the three nano-composites under 40 kV/mm for 1 h. This indicated that the nano-SiO_2_ particles could suppress the transfer of space charge in the material. 

**Figure 5:**
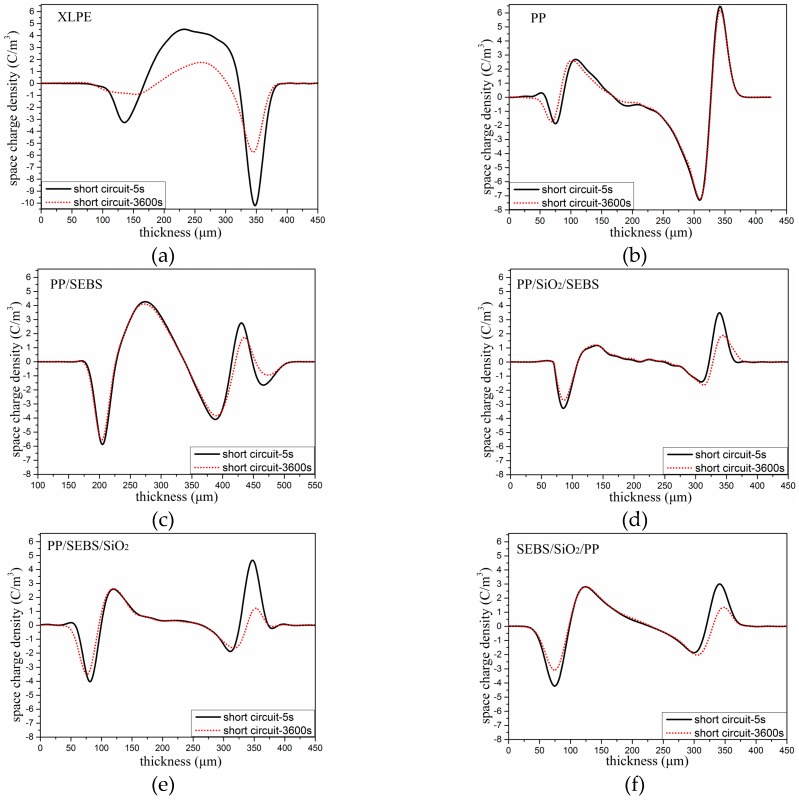
Space charge distribution under short circuit. (**a**) XLPE; (**b**) PP; (**c**) PP/SEBS; (**d**) PP/SiO_2_/SEBS; (**e**) PP/SEBS/SiO_2_; and (**f**) SEBS/SiO_2_/PP.

The space charge distribution of the samples under short circuit at 5 s and 3600 s are presented in the Figure 5. In order to further study the influence of SEBS and nano-SiO_2_ on the PP space charge, we calculated the space charge density of the samples by integrating the short circuit data of 5 s. The linear average space charge density *Q*(*t*) of the samples was obtained by dividing the middle space charge amount of each sample by the thickness of the specimen [25].

(2) $$Q(t)=\frac{1}{x_{2}-x_{1}}\int_{x_{1}}^{x_{2}}\rho (x,t)\mathrm{dx}$$

where *x*_1_ and *x*_2_ are the positive and negative electrode positions, respectively. *ρ*(x,t) is the space charge profile obtained at the short circuit data of 5 s.

The average space charge densities under short circuit at 5 s and 3600 s are shown in Table 3. It can be seen from the table that the average space charge density of XLPE was the highest under the short circuit at 5 s compared with those of the other materials. The space charge density decreased obviously after adding nano-SiO_2_ into PP/SEBS. After 3600 s, the space charge obviously decayed in XLPE, and the average space charge density in XLPE changed from the highest to the lowest, whereas the space charge in PP and its composites showed no obvious change. This shows that the trap depth of XLPE was shallower than that of PP.

### 3.5. Thermally Stimulated Depolarization Current 

**Figure 6:**
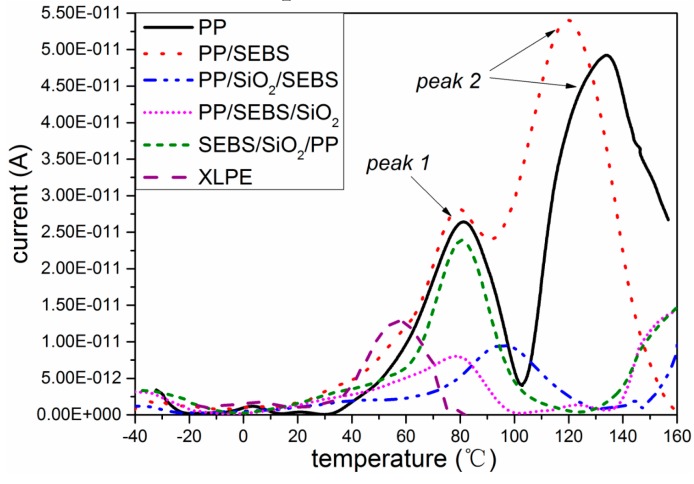
The thermally stimulated depolarization current (TSDC) pattern of XLPE, PP, and the PP composites.

The TSDC curves of all the samples are shown in Figure 6. The TSDC spectrum of XLPE has only one current release peak, and the peak position was at about 55 °C. Ieda’s research showed that the carrier trap corresponding to this temperature was mainly due to some structural defects in the polyethylene crystal region [26]. The depth of this trap was relatively shallow, and it resulted in the space charge easily migrating into the sample and decaying at a faster rate, as shown in Figure 4a and Figure 5a.

As the TSDC curves of PP and its composites show in Figure 6, the two peaks were corresponded to the α relaxation process in PP [27]. The relaxation peak at low temperature, which was named peak 1, was mainly derived from the movement of amorphous linked molecular chains and loose coils between lamellas, and part of the trap charge bound by this area was released. The molecular chain movement in the amorphous region was due to the internal strain of the amorphous phase and the space hindrance caused by the presence of the crystalline phase [27,28]. The high temperature peak, which was named peak 2, was the crystallization pre-melting peak, which originated from the movement of the molecules in the crystalline region. Ions and electrons usually migrate between molecular chains [13].

As shown in Figure 6, it can be seen from the curves that the current release peaks of PP were 26 and 49 Pa at 80 and 140 °C, respectively. After the addition of SEBS, peak 1 of PP/SEBS appeared at 78 °C, and the peak value of peak 1 increased. The peak position of peak 2 in the PP/SEBS composite decreased to 120 °C, and the peak value increased to 54 Pa. In the TSDC measurement, the current released at high temperature corresponded to a deeper energy level [29]. When SEBS was blended into PP, as shown in Figure 1, SEBS destroyed the PP crystal structure and introduced a large number of interface phases between SEBS and PP. As SEM and DSC test results showed, compared with PP, the *T*_m_ of PP/SEBS decreased, and the regularity of crystallization worsened; therefore, the current release peaks were likely to occur at low temperature. 

When nano-SiO_2_ was added into the polymer, the TSDC value of the three nano-composites was obviously reduced, which implied that there was a small amount of charges accumulated in the three nano-composites samples compared with PP. This phenomenon was also illustrated by the space charge test results as shown in Figure 5. Many studies have proven that nano-particles can influence the trap density, and the deep trap mechanism may suppress carrier migration [30,31]. When nano-SiO_2_ was introduced, a lot of deep traps around the interface between nano-SiO_2_ and PP were formed, and the positive and negative charges that would otherwise move freely in PP were bound in a nearby nano-SiO_2_ [31]. When the electric field was applied, the deep traps near nano-SiO_2_ could prevent the transfer of charges and suppress the formation of charges accumulated in the samples [31,32,33]. 

Comparing the TSDC curves of the three nano-composites, the peak value of the TSDC current in the SEBS/SiO_2_/PP nano-composite near 80 °C was significantly higher than those of the other two nano-composites, indicating that there was more space charge accumulation between lamellas in the SEBS/SiO_2_/PP nano-composite. The space charge test results also show that there was more space accumulated in the SEBS/SiO_2_/PP nano-composite. From the results of the SEM images, it was determined that nano-SiO_2_ in SEBS/SiO_2_/PP was more distributed in the SEBS phase, whereas nano-SiO_2_ in the PP phase was less distributed, which may be the reason for the decrease in the space charge suppression effect. It also can be seen from the TSDC graph that the current values of the three nano-composites still had an upward trend at 160 °C, and it was likely that a current peak would appear in higher temperature. Because the temperature was already higher than the melting temperature of PP, the current after this temperature was more likely to come from the release process of the trapped charge of nano-SiO_2_, suggesting that nano-SiO_2_ can form deeper trap levels.

### 3.6. DC Breakdown Strength

**Figure 7:**
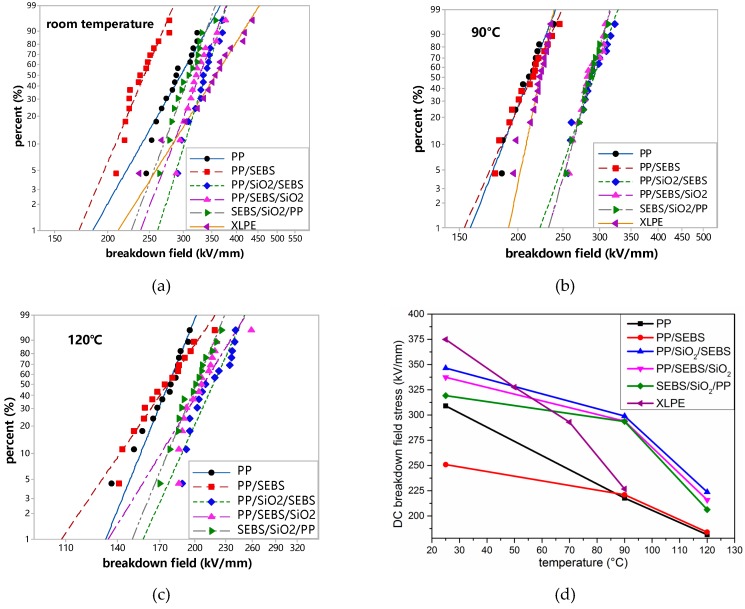
Weibull distribution of breakdown strength for PP and its composites. (**a**) Room temperature; (**b**) 90 °C; (**c**) 120 °C; (**d**) breakdown strength of materials at different temperatures.

The electrical breakdown strength of XLPE, PP, and the PP composites was analyzed by Weibull statistics, and the data were shown in Figure 7. The highest long-term operating temperature of the HVDC XLPE insulation was only 70 °C; therefore, the highest test temperature for XLPE was 90 °C in this study.

It can be seen from Figure 7 that XLPE had the highest breakdown field strength at room temperature, while the breakdown field strength decreased rapidly as the temperature increased. The breakdown strength of XLPE at 70 °C was about 290 kV/mm, and when the temperature increased to 90 °C, the breakdown strength of XPLE decreased to 230 kV/mm. For PP and its composites, it can be seen from Figure 7d that the breakdown strength of PP/SEBS was significantly decreased compared with PP at room temperature. This is mainly because SEBS was a thermoplastic elastomer, and its breakdown strength was lower than that of PP. In addition, it can be seen from the DSC and SEM results that SEBS also destroyed the crystal regularity of PP and also reduced the breakdown strength. As the temperature increased gradually, the breakdown strength of PP decreased significantly, while the breakdown strength of PP/SEBS decreased less. The breakdown strength of PP and PP/SEBS were quite close at 90 °C. The crystallization of the PP phase was gradually melted, resulting in a significant decrease in the breakdown strength of the PP phase. In addition, an aromatic hydrocarbon structure existed at the end of the SEBS macromolecular chain, which is likely to have a function as a voltage stabilizer and to improve insulation properties. The breakdown strength of the three nano-composites were obviously increased. Many scholars believe that when nano-particles are added into a polymer, a certain amount of deep traps appear in the interfaces, which can capture the space charge and reduce the carrier mobility in the interior material, and this results the improvement of DC breakdown strength [7]. It has been confirmed in the TSDC test above nano-SiO_2_ made the deep trap mechanism. Nano-SiO_2_ increased the depth and density of the traps inside PP, which enhanced the chance that the trap captured carriers. The breakdown field strength of PP/SiO_2_/SEBS was larger than that of the SEBS/SiO_2_/PP nano-composite. A possible reason for this was that the dispersion of nano-SiO_2_ in the SEBS phase was not uniform, and more nano-particles accumulated in the interface of SEBS and PP as shown in Figure 1. Nano-particles need to be uniformly and sufficiently dispersed in the polymer matrix material to produce significant electrical property improvement effects. When most of the nano-SiO_2_ particles were dispersed at the interface between the SEBS and the PP phases, the amount of nano-SiO_2_ particles in the PP phase and the SEBS phase was insufficient, and the electrical properties of the PP and SEBS phases could not be effectively improved. On the other hand, the carriers could obtain greater mobility and free paths in the SEBS phase, causing a decrease in electrical properties.

By comparing the breakdown strength of 500 kV XLPE materials and PP/SiO_2_/SEBS nano-composites, it was found that the breakdown strength of the self-made nano-composites at 90 °C was higher than that of 500 kV XLPE materials at 70 °C. Thus, 70 °C is the limitative operating temperature of 500 kV XLPE, which implies that the self-made nano-composite has a higher operating temperature.

## 4. Conclusions 

The dispersion characteristics of nano-particles in ternary composites composed by nano-particles, SEBS, and PP can be effectively controlled by changing the masterbatch system. The effect of nano-particle dispersion characteristics on the DC performance of ternary composites was more obvious than that on the mechanical properties. For PP/SiO_2_/SEBS, more nano-SiO_2_ was dispersed in the PP phase, and the optimal space charge suppression effect and the highest DC breakdown strength were obtained. The TSDC results confirmed that the traps in the composites were closely related to the structural defects in the PP phase, and the deep traps introduced by these nano-particles can effectively suppress the formation of space charge so that the electrical properties of PP/SiO_2_/SEBS were better than those of the other two composites. It was found that when the nano-SiO_2_ particles were more dispersed in the SEBS phase, the expected electrical property improvement was not obtained. Scanning electron microscopy showed that the nano-SiO_2_ particles in the SEBS phase were more dispersed at the interface than in the SEBS matrix, indicating that nano-SiO_2_ was poorly dispersed, which may be a reason why the electrical properties of the composite system were not significantly improved. The horizontal comparison with the DC performance of 500 kV HVDC XLPE materials showed that PP/SiO_2_/SEBS nano-composites have better space charge suppression and higher DC breakdown strength at 90 °C, which means that such nano-composites have good potential for use as recyclable HVDC insulation materials with engineering application value.

## Figures and Tables

**Table 1:** Abbreviation and component of the samples.

Sample	PP (phr)	SEBS (phr)	Nano-SiO_2_ (phr)	Blending Order
PP	100	0	0	-
PP/SEBS	75	25	1	masterbatch 1
PP/SiO_2_/SEBS	75	25	1	masterbatch 2 + SEBS
PP/SEBS/SiO_2_	75	25	1	masterbatch 1 + SiO_2_
SEBS/SiO_2_/PP	75	25	1	masterbatch 3 + PP

**Table 2:** Thermal parameters for the crystallization and melting processes.

Sample	*T*_c_ (°C)	*T*_m_ (°C)	*X*_c_ (%)
PP	116.2	165.8	46.22
PP/SEBS	113.2	164.2	46.23
PP/SiO_2_/SEBS	113.5	165	44.69
PP/SEBS/SiO_2_	113.8	164.6	44.56
SEBS/SiO_2_/PP	114.4	164.3	43.68

**Table 3:** Average Space charge density under short circuit at 5 s and 3600 s.

Time (s)	Average Space Charge Density (C/m^3^)					
	XLPE	PP	PP/SEBS	PP/SiO_2_/SEBS	PP/SEBS/SiO_2_	SEBS/SiO_2_/PP
5	3.01	2.16	2.59	0.73	0.83	1.35
3600	1.34	1.52	2.43	0.72	0.81	1.34
